# Educational and knowledge gaps within the European reference network on rare endocrine conditions

**DOI:** 10.1530/EC-20-0480

**Published:** 2020-11-30

**Authors:** Violeta Iotova, Camilla Schalin-Jäntti, Petra Bruegmann, Manuela Broesamle, Natasa Bratina, Vallo Tillmann, Olaf Hiort, Alberto M Pereira

**Affiliations:** 1 Endo-ERN Work Package ‘Education & Training’ Paediatric Chair, Department of Pediatrics, Medical University of Varna, Varna, Bulgaria; 2 Endo-ERN Work Package ‘Education & Training’ Adult Chair, Endocrinology, Abdominal Center, University of Helsinki and Helsinki University Hospital, Helsinki, Finland; 3 Endo-ERN Work Package ‘Education & Training’ European Patient Advocacy Group (ePAG) representative co-chair, Endo-ERN, Leiden, The Netherlands; 4 Department of Endocrinology, Diabetes and Metabolic Disorders, University Medical Center, University Childrens Hospital, Ljubljana, Slovenia; 5 Children’s Clinic, Tartu University Hospital, Tartu, Estonia; 6 Endo-ERN, Division of Paediatric Endocrinology and Diabetes, Department of Paediatric and Adolescent Medicine, University of Lübeck, Lübeck, Germany; 7 Endo-ERN, Division of Endocrinology, Department of Medicine, Leiden University Medical Center, Leiden, the Netherlands

**Keywords:** education, knowledge gaps, health care professionals, rare endocrine diseases, Endo-ERN, network

## Abstract

**Objective:**

The European Reference Network on Rare Endocrine Conditions (Endo-ERN), operational since 2017, consists of 71 health care providers (HCPs) in 19 EU member states. Our objective was to assess education and knowledge on rare endocrine conditions.

**Design and methods:**

A survey was developed and sent through the DIGIT-EUROSURVEY system to all Endo-ERN HCPs.

**Results:**

Response rate was 55% (*n* = 146), 95% physicians, 58% >20 years of experience, 96% academics. Largest knowledge gaps were reported for the transition and neonatal ages, and for the GPs. Less than 50% of HCPs had structured educational rare diseases (RD) plans, while 86% used RD specific guidelines. HCPs would share educational materials within Endo-ERN (74%), and participate in an accreditation model (85%). E-learning portals of the endocrine scientific societies used 58% (ESPE) and 64% (ESE). Most participants (90%) regarded Endo-ERN coordinated educational activities (annual meetings slots, webinars, etc.) as highly important and supported a common educational platform. Social media was perceived as important for educating patients (86%) but not for physicians (36%). Seventy-five % had developed patient education materials; only 31% had specific children’s materials, and by-country availability varied from 0 to 100%. Respondents provided newly diagnosed patients with their own material in the national language (81%); referred to advocacy groups (68%), and relevant online sources (50%). Respondents believed the European Commission should fund education through Endo-ERN.

**Conclusion:**

Identified knowledge gaps in rare endocrine disorders set the basis for fast catch-up through collaboration, alignment with patients’ needs, and further development of existing and newly developed educational resources.

## Background

Patients with rare disorders (RD) are few for any given country, while the required care is more complex in nature compared to common chronic disorders (1). RDs are characterized by unmet educational needs and knowledge gaps, lack of universal diagnostic tools and complex management. The RD patient community is very much aware of this and demands the rapid improvement of care (www.eurordis.org). Nowadays, people with RD live longer, and children who might have not survived in the past need decent care throughout their lifespan (2). The European Reference Networks for rare diseases were established and began operating in March 2017, covering large geographical parts of Europe and, by definition, collecting best expert knowledge. The European Commission’s (EC) concept was primarily to reduce health care inequalities among member states, such as different health service and care development, discrepancies between health funds and care availability, but most of all because of the unlikely scenario that all countries could develop and provide the best expertise for rare and ultra-rare disorders (3). Networks were thus expected to improve cross-border health care, the impact of inspirational leaders and network governance being crucial.

From the beginning, the biggest challenge for the Networks was the lack of additional funding and uncertainties regarding national/local support, despite 11 predefined ‘system levers’ on integrated care frameworks (https://ec.europa.eu/health/sites/health/files/systems_performance_assessment/docs/2017_blocks_en_0.pdf). Nevertheless, the interest among health care providers (HCPs) was enormous. To clarify, the term HCPs stands for hospitals and other health establishments. The number of ERNs at the initiation of this very ambitious European project was 24, with more than 300 member HCPs including 900 expert units. Main requirements for ERN membership were RD expertise established at the national level, and multidisciplinary life-long care (3). The Networks are now in their 4th year, and various successful examples and positive changes in the landscape of RD in Europe are appearing. Moreover, on an on-going annual basis, the EC audits Network members, performs random site visits to check the accuracy of self-assessment documents, and has developed a continuous monitoring program for ERNs, that includes mandatory periodic reporting on 18 general key performance indicators.

A characteristic feature of rare endocrine diseases is the differing spectrum of disorders and patients encountered in pediatric compared to adult age range. In childhood, there is a large variety of genetic conditions, and many patients still die early because of delayed diagnosis or nonexistent treatment (4). In adulthood, patients with rare endocrine diseases are grouped in fewer entities, but in larger numbers. In-between, the age of transition should be covered by pediatric endocrinologists and endocrinologists working together. These characteristics were taken into account when creating the structure of the European Reference Network on Rare Endocrine Conditions (Endo-ERN), which has 8 Main Thematic disease Groups (MTGs) (https://endo-ern.eu/specific-expertise/overview-mtg/). Every MTG has 4 chairs – a pediatric, and an adult professional, and a pediatric and an adult patient advocacy group (ePAGs) representative. The specific activities that the Network should fulfill for the initial 5 years of existence were defined ‘horizontally’ in Work Packages (WPs) – WP1: Education and Training, WP2: E-health and ICT, WP3: Research and Science; WP4: Quality of care and Patients’ views and WP5: Diagnostics and Laboratory Analysis. Every WP also has four chairs as described previously. As required, Endo-ERN Network is chaired by an adult endocrinologist that fully shares the activities with a pediatric co-chair, assuring consensus between parties. All chairs constitute the Steering Committee (SC). This structure was endorsed by both major European professional societies, ESPE (European Society of Pediatric Endocrinology) and ESE (European Society of Endocrinology).

As stated in the Endo-ERN network application, the mission is to reduce and ultimately abolish inequalities in care for patients with rare endocrine disorders in Europe, by facilitating knowledge sharing, health care and research. One of the first formulated and adopted tasks for *WP1 Education and Training* was to assess the educational and knowledge gaps as well as map existing resources on rare endocrine conditions, by performing a survey among the Network members, the results of which are presented here.

## Methodology

First, a representative WP1 Working group with two participants (one pediatric and one adult) from all eight MTGs was established. A maximally transparent procedure was followed by offering all members the possibility to participate, and those expressing interest were invited. The principles of equality by specialty (adult or paediatric), gender, and country/European region were taken into account when composing the final members of the Working group. All eight MTGs were represented, 56% of the participants were female; 50% were pediatric and 50% adult endocrinologists.

The first draft of the Survey was created by the WP1 Chairs. The rationale was based on the principles for assessing education and training (5) and the identification of gaps in:

– Knowledge
– Competence
– Performance

Compliance with the specifics of the Network was emphasized – equal representation of pediatric and adult needs, and sensitivity to patient views and demands. The first survey was addressed to health professionals (HPs). To clarify, ‘HPs’ stands for health professionals (doctors, nurses, etc.). We also assessed existing resources, local and international, self-created and translated patient and HPs educational materials, as well as readiness to share within and contribute to the Network.

To overcome some of the difficulties in developing a survey (diversity in the level of development of the member HCPs, type of diseases cared for and their rarity, some being ultra-rare, language and cultural traits), WP1 Chairs first created a basic, universal short survey, which was tested for feasibility and validation within the WP1 Working group.

### Feasibility and validation procedures

The feasibility/validation process took place in August/September 2017. WP1 Working group members (*n* = 20) answered the Survey questions and reported on any identified inconsistencies/problems. Ten answers (50%) were received. Through this process, mistakes, repetitions and missing variables, as well as unclear expressions were identified and corrected. The most important results of the feasibility/validation survey were then presented at the SC Meeting in October 2017. The final Survey consisted of 36 questions, and was approved by the SC (Supplementary Fig. 1, see section on supplementary materials given at the end of this article). The DIGIT-EUSURVEY instrument was chosen for its known reliability, possibilities to survey many participants and reliable statistic output. It takes around 20 min to complete the survey.

### Data collection and analysis

The survey was distributed on 1 November 2017 to all HCPs representatives of Endo-ERN. The accompanying e-mail explained the pre-requisite of a response from at least one member per MTG for the given HCP. Since most HCPs participate in more than one Main Thematic Group (MTG), the maximum expected number of respondents was 268 (71 HCPs, multiplied by the number of participating MTGs within Endo-ERN). Three reminders were sent, the last one on 2 December 2017. The Survey was closed on 1 January 2018.

Analysis was done automatically with the DIGIT-EUROSURVEY system.

## Results

### Basic characteristics and demographics of the respondents

**Figure 1:**
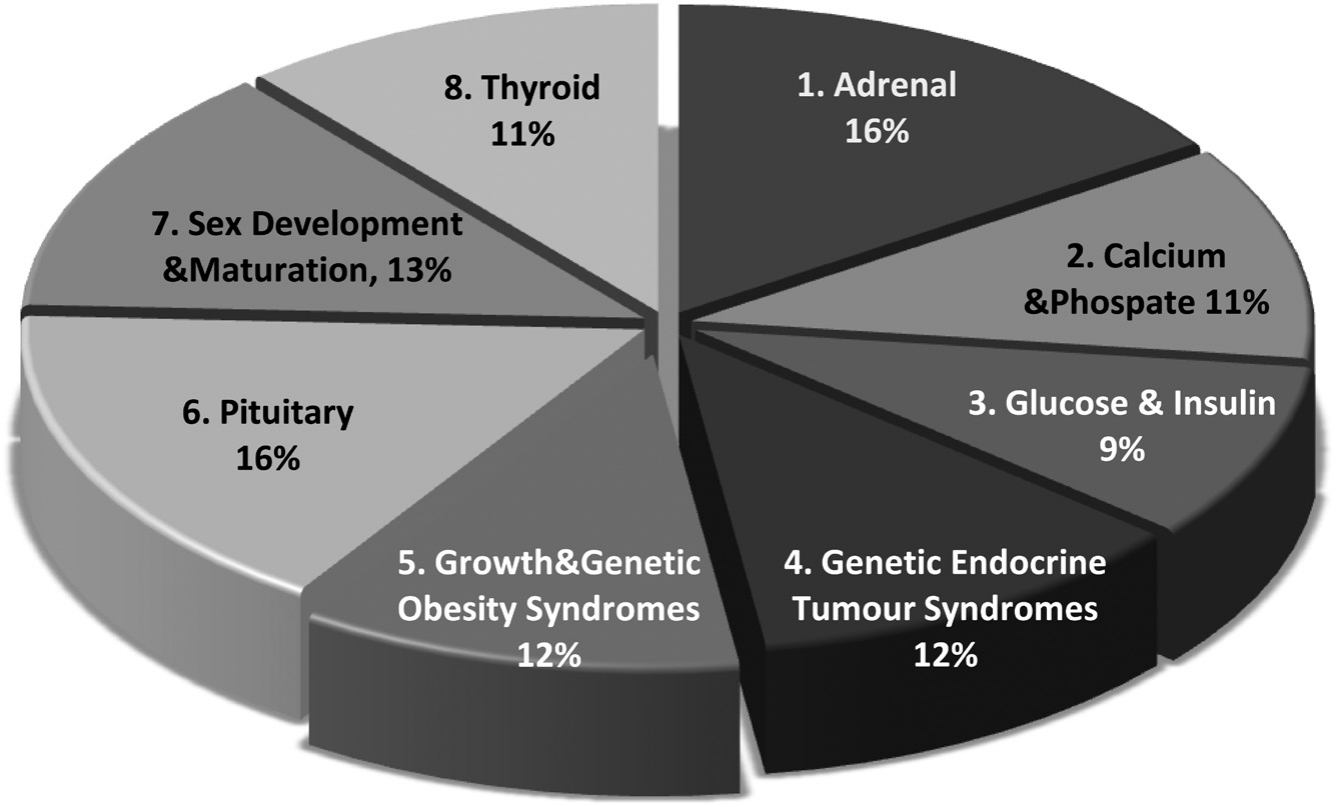
Distribution of HCPs respondents by membership in one of the eight main thematic groups (MTGs) of the Endo-ERN.

A total of 146 (55%) responses were received from all 19 member countries. The three most represented countries were Italy (*n* = 31), The Netherlands (*n* = 22) and UK (*n* = 17) while Luxemburg and Portugal participated by one responder each. Respondents were predominantly physicians, with less than 5% other HC professionals, 53% were female and 96% represented University hospitals. Of the respondents, 36% were pediatric, 59% adult specialists and 5% others. Most participants were >40 years (81%), and 58% had >20 years of practice with RD. The represented HCPs were evenly distributed between all MTGs (Fig. 1).

MTG6, pituitary, (20%) and MTG1, adrenal (19%) were slightly overrepresented among the individual responses, followed by MTG3, Genetic disorders of glucose and insulin homeostasis and MTG7, Sex Development and Maturation (12% each).

### Knowledge gaps

**Figure 2:**
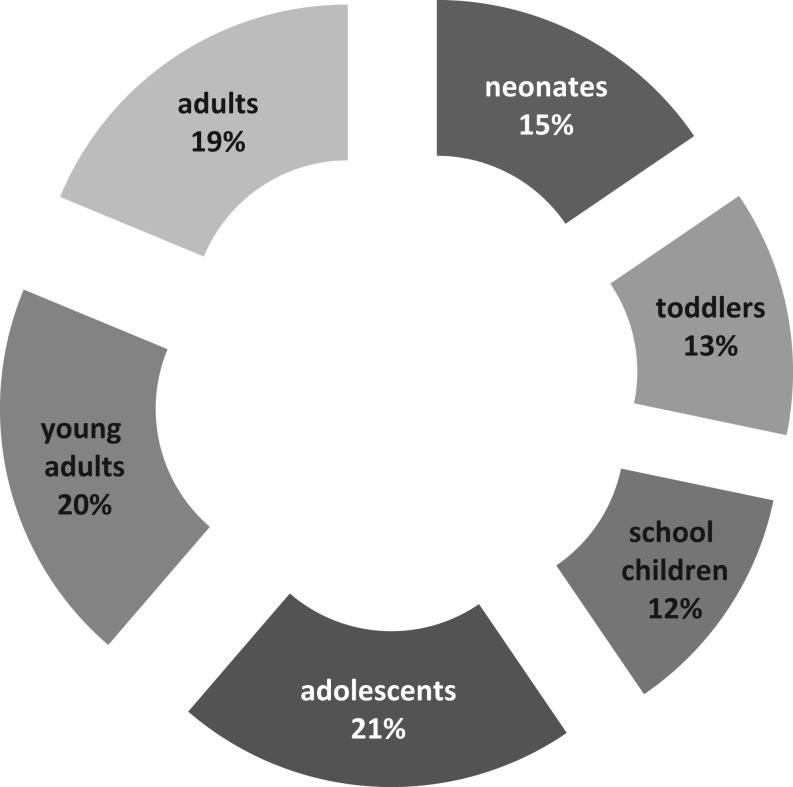
Patient age interval least covered by enough knowledge.

The largest knowledge gaps in patient care were reported for adolescent, young adult (transition), and adult patients (Fig. 2). When analyzed according to the respondent’s specialty, pediatricians identified additional knowledge gaps regarding the neonatal age (49%).

The largest knowledge and training gaps in the field of RD among physicians were reported for GPs (71%), followed by students and medical specialist trainees (61% each), and specialists (51%). Only 45% of the HCPs had a structured RD educational plan, aimed at any kind of medical staff; 36% reported a specific training program for GPs. Health personnel’s RD knowledge was periodically assessed through structured programs by 53% of the HCPs.

### Educational and practice needs of the physicians

The importance of different diagnostic and treatment-related aspects of RD as rated by doctors were as follows: disease diagnosis: 99%; disease follow-up issues: 99%; available treatments: 99%; disease prognosis: 95%; advice from centers of excellence: 92%; local health care organizations: 83%; cross-border health care: 70%.

Most of the HCPs developed and used national/local RD disease-specific guidelines, which are publicly available (86%); 30% had already collaborated with other Endo-ERN members to develop/translate/adapt guidelines. A considerable proportion of the HCPs reported to be ready to share educational materials within the Endo-ERN (74%), and to participate in the creation and application of an accreditation model (85%). Only 12% had a fully available repository of educational materials, including media coverage and lay public activities, and 27% had access to partial archive only. Intriguingly, 95% were ready to collect such materials in the future, and 72% were ready to share these within Endo-ERN.

### Educational tools for physicians

Of the online available educational tools, 58% of the respondents (96% of pediatric and 33% of adult endocrinologists) consider the ESPE e-learning portal as more appropriate to develop as a RD learning tool, and 64% (25% of pediatric and 90% of adult endocrinologists) consider the ESE e-learning platform as more appropriate. Other resources were used less often. In their daily practice with RD, HPs used and most often recommend trainees to use the OMIM NLM online database (>76%) and Orphanet (>75%). Social media were assessed by HPs as very important for the education of patients (86%), but less important for that of physicians (36%).

A unanimous wish was to have special RD boosting knowledge slots at the ESE/ESPE annual meetings, and dedicated postgraduate courses (86% each). Almost all answers (95%) were in favor of a common RD educational platform, uniform for the Network. The large majority of participants (90%) supported advertising different types of educational activities (webinars, pod-casts, lectures, journal clubs) through Endo-ERN.

### Educational needs of patients as perceived by doctors

Survey participants rated the following items as most important for patients: available treatments (99%); prognosis (94%); rehabilitation and everyday issues (94%); nature and course of the disease (88%); psychological aspects (85%); social and economic issues (68%).

Regarding educational materials for patients, 75% of respondents had developed such, 55% of which were made publicly available, and 30% had specific educational plans for ePAGs. HCPs less frequently (31%) developed/used specific educational materials for children. Importantly, the by-country availability of educational materials varied from 0% to 100%.

When diagnosing a new patient with RD, respondents usually provided their own material in the national language (81%); printed available online material in the national language (37%), or in other languages (18%); In addition, patients were referred to PAGs (68%), and the patient/family were also referred to relevant websites/social media sources (50%).

### Financing of educational activities

Opinions on how educational activities should be financed were assessed on a Likert scale. ‘Likely’ and ‘very likely’ future educational Endo-ERN funding possibilities were: EC funding through Endo-ERN (86%); EC educational calls specific for the ERNs (73%); pharma activities and governmental/state activities (51% each); HCPs funding their own activities (45%); medical schools funding (42%); ePAGs raised funding for education (32%).

The answer to the question whose responsibility it is to finance educational activities was in the vast majority of respondents that this should be EC funded work, through Endo-ERN, while only a few respondents answered that this could be the responsibility of the state/government, or that of Medical schools.

## Discussion

For rare diseases, quality education achieved through diverse actions, acknowledging the individual needs of the different conditions is pivotal for sustainable health care systems (6). Professional education is mandatory for prompt diagnosis and proper management of RD. At the same time, patients need to understand the condition in order to secure best possible care and respond optimally to it. Our aim was to map the current situation and knowledge on rare endocrine conditions in Europe through detailed surveying core specialists from Endo-ERN reference centers. The approach was to collect as much as possible groups-of-conditions specific information, assuring maximal representation of educational needs as defined by the highest level of medical health professionals. Extensive clinical experience - also called ‘hands-on education’ (7, 8) is more important for professional development than the formal training (9). In the present study, the age of the respondents (>40 years), and specifically their long-standing academic experience (>20 years) are representative of the validity of the collected information.

Some inequality existed in the adult and pediatric representation of the respondents, most probably due to more adult representation of specialists from the two MTGs (pituitary and adrenal) with the highest response rates. Alternatively, there is a possibility that less pediatric endocrinologists were addressed by the HCPs. Consequently, to correct for potential bias, additional analyses were performed according to the profession of the respondents. As expected, the results differed only in the preference of educational tools (ESPE e-learning for pediatric endocrinologists and ESE e-learning platform for adult endocrinologists), as well as in which area the respondents reported the lack of sufficient knowledge (neonatology for pediatric endocrinologists, and adolescence and young adulthood for both specialties). To our mind, these findings further underpin the importance of networking in order to reduce existing gaps.

Difficulties to access specific knowledge and competences concerning adolescence/young adulthood are not limited to endocrine diseases or RD only. Reported evidence on successful transitional health care is limited, and available written educational protocols are hardly available (10, 11). The evaluation of the knowledge and practice educational interventions is difficult even in case of existent robust outcomes (11), such as HbA1c in patients with diabetes (10). Even if well planned, controlled and documented, educational interventions are often not directly adding value to usual care (6). Understandably, every HCP is managing the process within its own healthcare system and accessible means, which justifies alignment efforts from the Network.

A recent Belgian study addressing educational needs of GPs (12) showed that most of them already used various RD information channels. However, their educational endeavors were prompted only by direct encounter with RD patients. Most of the respondents identified better penetration of RD education in the academic medical curricula as a major lever for improvement of RD knowledge, as well as the introduction of so-called ‘red flags’, drawing attention to, and increasing awareness of a possible RD in the clinical practice. However, the participants in this study (12) were not directly involved in expert RD care which sharply contrasts with the participants of the current study. It is thus reassuring that expert HPs identify the same gaps in RD education, and suggest similar approaches. One positive example is the ESE PARAT program – an initiative to uncover and address unmet educational and scientific needs in parathyroid disorders that also represent rare diseases (13). Another interesting example is the report presented by Cismondi *et al.* (14). The paradigm of single rare metabolic disease care was discussed during a workshop, concluding that efforts for improving care should begin with education, ultimately leading to the successful achievement of all goals. On the other hand, a study among Polish medical students showed not only a lack of knowledge but also a lack of interest in RD (15). Mathew formulates a few useful principles to be taught to students that enable them not to miss a rare condition while collecting history and examining a patient (16).

In the present study, the respondents considered correct diagnosing of RD as the most important task for them as medical professionals, while knowledge on available treatments and prognosis were considered most important for the patients. Social media was not viewed as an educational tool for doctors. As pointed out by Heon-Klin (17), the Networks need virtual cross-border highly-specialized health care advice services to overcome fragmentation and help HPs in their work with individual entities/patients. This has been implemented now by all ERNs by means of the Clinical Patient Management System (CPMS). CPMS allows for easy and safe patient consultations by multidisciplinary teams, and as such, also serves as a powerful educational tool providing direct access to the best expertise in Europe. Through CPMS, the ‘tacit knowledge’ (acquired through self-experience and high volume work) finds easy ways to merge with validated ‘explicit knowledge’ into higher competence (18), thus potentially improving RD care in a more efficient way based on cooperation.

According to the results of the present study, and in line with the literature, the time has come for structured theoretical and practical education on RD at all levels – for medical students, during postgraduate training and as continuous medical education in everyday practice. At the beginning of the ERNs, a major uncertainty was how to finance these programs. This uncertainty and hesitancy are also reflected in the current early survey. The only unconditional result is that educational activities must receive funding (100%), and >85% accentuated that this should be distributed through the Endo-ERN. Iskrov *et al.* (17) proposed establishing postgraduate training programs devoted to RD, and provided alternative suggestions for financial stability.

Hiort *et al.* (19) presented experience from the educational activities of previous scientific actions with European financial support - the COST Actions DSDnet and GnRH Network, using specific instruments such as early career researchers short scientific missions and training schools. In addition, they defined the way-forward for the ERNs to develop activities and extensive use of the European Joint Program Co-fund on RD research, education, and training (https://www.ejprarediseases.org/index.php/training-and-empowerment/e-learning/). The task is very challenging and there is compelling evidence that this cannot be executed outside the Expert centers/respective Networks. A recent German study (20) tried to attract interest among physicians outside an expert center to metabolic RDs, offering education and mutual supported care for these patients with a formal contract, and supplying informational materials and yearly training. Intriguingly, although many of the invited physicians had already referred patients to this center, only 0.5% of them agreed to collaborate (20). The main reason not to participate was the expected very low number of patients at the doctors’ own practices.

A large study among Australian pediatricians caring for RD patients demonstrated that most participants felt unprepared for the challenges of such care (3). They desired one online portal through which the doctor could access many resources, similar to the results of the current survey. The participants in Endo-ERN prioritize a joint educational platform for RD, and accept streaming of knowledge through the Endo-ERN. Further collaboration with ESE and ESPE as well as possible links to the already existing ESE and ESPE e-platforms is important. Some of the requirements, such as slots at the scientific professional societies’ annual meetings and Endo-ERN webinars, have already been established. However, programs/instruments for knowledge and training/education assessment are still lacking universally (21), as well as specific patient materials (especially for children) and funding of activities. Fund-raising is something that patient organizations are doing regularly. It seems that health care professionals should also not refrain from such activities (6). Increased funding for education and training at this historical moment for RD in time should be pursued since the gathering of expertise within the Networks means boosting of knowledge and practice. Recent developments in the integrated care for RD stress that patients and their families should be directly engaged in decisions about their care (1), the basis of which again is knowledge.

Limitations of the current study include the possibility of specific language problems among respondents that could have resulted in some inconsistencies in answers. Assessment of patients’ educational means as perceived by HCPs has its limitations. WP1 has therefore recently performed another large survey among endocrine RD patients all over Europe (unpublished).

Strengths of the study include the highest possible experience of the respondents, as they are nationally endorsed for specific expertise senior health professionals to manage patients with rare endocrine disorders. With 50% participation rate in the feasibility study where only highly motivated MTGs members took part, the response rate of 55% in the general survey was better than expected. Equal representation by profession, country, MTG, etc., the guaranteed validity of the results. We demonstrate that from the beginning, the approach and expectations of the respondents are similar regarding the educational and training aspects of the Network.

In conclusion, educational goals expected to be achieved by the Network include the transition and neonatal age, as well as assessment of and addressing the educational needs of patients. The identified knowledge gaps in rare endocrine disorders set the basis for their prompt closure through collaboration, alignment with patients’ needs and further development of existing educational platforms such as ESE and ESPE online learning tools and establishment of specific Endo-ERN resources that are underway.

## Supplementary Material

ENDO-ERN WP 1 Questionnaire

## Declaration of interest

The authors declare that there is no conflict of interest that could be perceived as prejudicing the impartiality of the research reported.

## Funding

Endo-ERN is a European Reference Network co-funded by the European Union’s 3rd Health Programme (CHAFEA FPA grant No 739527).
