# Supplementary material for: Educational and knowledge gaps within the European reference network on rare endocrine conditions
Source: Endocr Connect. 2020 Nov 30;10(1):37–44. doi: 10.1530/EC-20-0480 (PMC7923050; doi:10.1530/EC-20-0480)
Supplement: ENDO-ERN WP 1 Questionnaire [file supplementary_material.pdf]

ENDO-ERN WP 1 Questionnaire

Fields marked with * are mandatory.

WP 1 Questionnaire 1

Dear colleague, dear Endo-ERN HCP representative,

This is a survey that is aimed at gathering initial information about existing sources and tools of education and teaching, related to rare endocrine diseases, as well as suggestions how to enrich those. The survey is created by the Working Group 1 Education & Teaching of the Endo-ERN (www.endo-ern.eu), and the results will be analyzed and presented at the next General Assembly of the Network (Feb, 2018).

Please, forward the survey to at least one representative of the Main thematic groups (MTGs 1 to 8), in which your HCP is participating. E.g. if your HCP takes part in 3 MTGs, we expect from you at least 3 filled in questionnaires.

Since this is the first survey, we apologize for its length! We believe you don't need a special preparation to answer all question, and it will take about 20-25 min overall. If the results are worth sharing with the general scientific and societal public, a paper will be prepared, acknowledging your contribution!

With best regards,

WP 1(education and training) co-chairs

Camilla

Violeta

Petra

Who is responding?

*1. Country

- Austria
- Belgium
- Bulgaria
- Croatia
- Cyprus
- Czech Republic
- Denmark
- Estonia
- Finland
- France
- Germany
- Greece
- Hungary
- Ireland
- Italy
- Latvia
- Lithuania
- Luxembourg
- Malta
- Netherlands
- Poland
- Portugal
- Romania
- Slovak Republic
- Slovenia
- Spain
- Sweden
- United Kingdom

*2. Gender

- Female
- Male

*3. Age

- < 20
- 21 - 30
- 31 - 40
- 41 - 50
- 51 - 60
- > 61

*4. Profession

- Physician
- Nurse
- Nutricionist
- Psychologist
- other Health Care Professional
- Other

4 a. If OTHER, please specify !

*5. Years in practice

- < 10
- 11 - 20
- 21 - 30

*6. Characteristics of the HCP

- University hospital
- Academic outpatient
- Public hospital
- Public outpatient
- Private hospital
- Private practice
- Other

6 a. If OTHER, please specify !

*7. Your HCP takes part in which of the following Main Thematic Groups (MTGs). Mark as many as needed.

- 1. Adrenal
- 2. Disorders of Calcium & Phospate Homeostasis
- 3. Genetic Disorders of Glucose & Insulin Homeostasis
- 4. Genetic Endocrine Tumour Syndromes
- 5. Growth & Genetic Obesity Syndromes
- 6. Pituitary
- 7. Sex Development & Maturation
- 8. Thyroid

*7a. You are answering for Main Thematic Group

- 1. Adrenal
- 2. Disorders of Calcium & Phospate Homeostasis
- 3. Genetic Disorders of Glucose & Insulin Homeostasis
- 4. Genetic Endocrine Tumour Syndromes
- 5. Growth & Genetic Obesity Syndromes
- 6. Pituitary
- 7. Sex Development & Maturation
- 8. Thyroid

*8. You work as

- [ ] a pediatric endocrinologist
- [ ] an adult endocrinologist
- [ ] mostly within transition of the young patients

1. Questionnaire

* 8. What online educational tools do you use regularly in your practice with rare diseases (you may choose more than one):

- [ ] OMIM
- [ ] Orphanet
- [ ] FindZebra.com
- [ ] NORD
- [ ] GARD
- [ ] Other

8 a. If OTHER, please specify !

*9. What online tools do you usually recommend to your trainees ?

- [ ] OMIM
- [ ] Orphanet
- [ ] FindZebra.com
- [ ] NORD
- [ ] GARD
- [ ] Other

9 a. If OTHER, please specify !

*10. Which of the existing e-learning tools (platforms) is appropriate for future development and specialization in the field of rare diseases education?

- ☐ ESPE e-learning portal
- ☐ ECE e-learning platform
- ☐ Other

10 a. If OTHER, please specify !

*11. Are you in favor of using ECE/ESPE annual meetings and postgraduate courses as platforms for boosting education about rare diseases, e.g. special slots in the program?

- ○ Yes, I do support more structured RD Program
- ○ No, there is enough penetrance of RD in the Programs

*12. Is there any regular/periodic educational assessment of doctors/health care professionals at your HCP?

- ○ Yes
- ○ No

12 a. If YES, please explain:

*13. In your view, major education and teaching gaps in relation to rare endocrine diseases exist in (you can choose more than 1 answer):

- ☐ Students' education
- ☐ Specialist trainees' education
- ☐ GPs' education
- ☐ Specialists' education
- ☐ Other health specialists education
- ☐ Patients' education
- ☐ The lay public
- ☐ Other

14 a. If OTHER, please specify !

* 14. In your opinion, which patient age interval is the least covered by enough knowledge?

- Neonates
- Toddlers and pre-school children
- School children
- Adolescents
- Young adults
- Adults

15. In your opinion, on the scale from 1 (very unlikely) to 5 (very likely), who should be the future funding body of the ERN educational activities?

	1 - Very Unlikely	2 - Unlikely	3 - Neither	4 - Likely	5 - Very Likely
* HCPs themselves					
* Medical schools					
* EC through Endo-ERN					
* EC through special calls for educational initiatives					
* Publically raised money with the help of PAGs					
* Pharma supported initiatives					
* States/governments					
* Other					

15 a. If OTHER, please specify !

* 16. Are you in favor of a common educational tool/platform for the Endo-ERN ?

- Yes
- No

17. On the scale from 1 (not at all) to 5 (very relevant), which information is most relevant to patients?

	1 - Not at all	2 - Slightly	3 - Moderately	4 - Relevant	5 - Very
* Nature and course of disease	☐	☐	☐	☐	☐
* Available treatments	☐	☐	☐	☐	☐
* Prognosis	☐	☐	☐	☐	☐
* Rehabilitation and everyday life issues	☐	☐	☐	☐	☐
* Psychological aspects	☐	☐	☐	☐	☐
* Social and economical aspects	☐	☐	☐	☐	☐

18. On the scale from 1 (not at all) to 5 (very relevant), which information is most relevant to doctors?

	1 - Not at all	2 - Slightly	3 - Moderately	4 - Relevant	5 - Very
* Disease diagnosis	☐	☐	☐	☐	☐
* Available treatments	☐	☐	☐	☐	☐
* Follow-up	☐	☐	☐	☐	☐
* Prognosis	☐	☐	☐	☐	☐
* Local health care organisation	☐	☐	☐	☐	☐
* Centers of excellence advice	☐	☐	☐	☐	☐
* Available transborder care	☐	☐	☐	☐	☐

* 19. Do you believe that social media have a role in the HPs' education?

- ○ Yes
- ○ No

* 20. Do you believe that social media have a role in the patients' education?

- ○ Yes
- ○ No

* 21. Do you have national/local guidelines for the diseases that are represented in your Main Thematic Group (MTG) ?

- ○ Yes
- ○ No

21a. If yes, for which diseases? (please, list)

*22. Are these (at least some of these) publicly available?

- Yes
- No

*23. Do you collaborate with another member of the End-ERN for developing/adapting/translation of specific guidelines?

- Yes
- No

23 a. If yes, please list !

*24. Does your HCP have a specific educational plan (strategy) of relevance to rare endocrine diseases?

- Yes
- No

*25. Have you created specific education materials for patients?

- Yes
- No

25 a. If yes, please list !

*26. Do you have educational materials specifically developed for children?

- Yes
- No

* 27. Are these (at least some of these) materials publicly available?

- Yes
- No

*28. Do you have specific educational plans for organised PAGs?

- Yes
- No

*29. Have you created specific education materials for health care professionals?

- Yes
- No

29 a. If yes, please list !

*30. Are you (Is your HCP) ready to share educational & teaching materials with other network members?

- Yes
- No

30 a. If yes, please, specify conditions if foreseen

*31. Would you participate in/approve creating an accreditation model for educational capacity of a HCP that is a member of the Endo-ERN?

- Yes
- No

*32. Are there any specific days for raising awareness/teaching about rare endocrine diseases that you mark/celebrate every year?

- Yes
- No

32 a. If yes, please list !

*33. Do you apply special strategy/plans to educate GPs?

- Yes
- No

33 a. If yes, please list !

*34. Do you have an archive/repository of media coverage, awards, lay literature publications, related to your educational activities?

- Yes
- No
- Partially

*34 a. If yes, would you share those with the ERN?

- Yes
- No

*35. Would you agree to collect materials prospectively if there is an easy and user-friendly way to do that?

- Yes
- No

*36. What do you usually do when you diagnose a new rare patient:

- Give out own material in the local language
- Print out web available material in the local language
- Print out web available material in other language
- Refer the patient to PAG
- Show the patient/family relevant web-sites/social media resources
- Other

36 a. If OTHER, please specify !

*37. What are your HCP's educational plans for 2018? Can you share?

*38. How do you think Endo-ERN could assist you?

*39. Are you in favor of simultaneous educational activities advertised through the Endo-ERN (webinars, pod-casts, lectures, journal clubs, etc.)?

- Yes
- No

*40. Would you mind sharing some of your educational activities/related diseases from the last 3 years (2015-2017):

Thank you very much !
